# *Yersinia enterocolitica* serotype O:9 cultured from Swedish sheep showing serologically false**-**positive reactions for *Brucella melitensis*

**DOI:** 10.3402/iee.v2i0.19027

**Published:** 2012-12-11

**Authors:** Erika Chenais, Elisabeth Bagge, Susanne Thisted Lambertz, Karin Artursson

**Affiliations:** 1 Department of Disease Control and Epidemiology, National Veterinary Institute, Uppsala, Sweden; 2 Deparment of Bacteriology, National Veterinary Institute, Uppsala, Sweden; 3 Research & Development Department, National Food Agency, Uppsala, Sweden

**Keywords:** brucellosis, false-positive reactions, Rose Bengal Test, Complement Fixation Test, *Yersinia enterocolitica*

## Abstract

In a herd of 20 sheep in Sweden, a country where brucellosis has never been diagnosed in sheep or goats, a total of six sheep were found serologically positive to *Brucella melitensis* in two different rounds of sampling. *Yersinia enterocolitica* serotype O:9 could at the time of the second sampling be isolated from four sheep, one of them at the same time serologically positive for *B. melitensis*. The article describes the case and gives some background information on brucellosis and *Y. enterocolitica* in general as well as a more specific description of the Swedish surveillance program for *B. melitensis* and the test procedures used. The problem with false**-**positive reactions, in particular its implications for surveillance programs in low prevalence or officially brucellosis-free countries, is discussed.

In many parts of the developing world, brucellosis is endemic with seroprevalence of up to 40% in cattle (1). Human brucellosis is one of the most common bacterial zoonotic infections worldwide and a yearly infection rate of 500,000 human cases is estimated (2, 3). *Brucella melitensis* is the principal cause of human brucellosis (4). Sadly, brucellosis is also classified as the most neglected human disease in terms of morbidity, socioeconomic effects, and financial support for research (5). The economic impact of brucellosis in animals can be devastating (6), especially in developing countries where control of diseases is scarce and animals have multiple roles, including constituting a substantial part of the social security net for other disasters (7). Brucellosis causes abortions and sterility in animals. The burden of human disease adds to the economic impact (6). Since the organism is shed in large numbers in the animal's urine, milk, and placental fluid, exposure to infected animals and animal products causes brucellosis in humans (8). It has been recognized that prevention of brucellosis in humans relies on the control and eradication of the disease in animals (2, 9). *Brucella abortus* has been eradicated in many European countries but *B. melitensis* is still endemic in the Mediterranean basin (10, 6). Efforts to eradicate brucellosis can be hampered by the fact that the serological screening tests widely used sometimes give rise to a large proportion of false**-**positive reactions (11). This is especially a problem in low prevalence countries or countries free of brucellosis. Brucellosis is by large an occupational disease with shepherds, abattoir workers, veterinarians, and personnel in microbiologic laboratories being over represented among the patients (4, 12).

## Brucellosis

Brucellosis is caused by gram-negative bacteria belonging to the genus *Brucella*. To date, the genus consists of 10 species, each having a preferred animal host (9). Of the 10 brucella-species so far recognized, *B. melitensis, B. abortus*, *Brucella suis*, and *Brucella inopinata* have high zoonotic potential. *Brucella canis* has a moderate zoonotic potential and the remaining species, *Brucella neotomate*, *Brucella ovis*, *Brucella ceti*, *Brucella pinnipedialis*, and *Brucella microti*, have no or unknown zoonotic potential (9). However, reports of *B. ceti* and *B. pinnipedialis* infecting humans are increasing and there are also reports of possibly two new *Brucella* species, one of them isolated from baboons (5). The main pathogenic species causing reproductive and abortive disorders in domestic animals worldwide are nevertheless *B. melitensis*, which occur mainly in sheep and goats, *B. abortus* in cattle, and *B. suis* in pigs (2, 13, 14). Despite different *Brucella* species being relatively host specific, *B. melitensis* emerges as an infection also affecting cattle (4) as does *B. suis* (2, 9). In 2012, outbreaks of *B. abortus* and *B. melitensis* in cattle have been reported from France, whereas *B. abortus* and *B. suis* were found in cattle in Belgium (http://www.promedmail.org/?p=2400:1000, http://www.oie.int/en). In animals, brucellosis mainly causes reproductive disorders such as abortions, orchitis, and epididymitis (14). Systemic signs and deaths are rare, except in fetuses and newborn animals. The period between infection and abortion or other reproductive signs is variable and the infection can be latent and transmitted in utero by clinically unaffected females (8). Apart from causing abortions, the infection reduces fertility, causes weight loss, and lowers the milk yield of animals (6). The typical signs in infected people are acute febrile illness with non-specific flu-like signs, such as fever, headache, malaise, back pain, myalgia, and generalized aches (6). However, brucellosis in humans can also be asymptomatic, and the course of the illness is extremely variable, the clinical signs may appear insidiously or abruptly (12). Some patients recover spontaneously, while others develop persistent symptoms that typically wax and wane (12). The mortality rate is low.

The bacteria can infect animals or humans via oral and respiratory routes as well as through intact mucous membranes and even intact skin (6). Following entry, the bacteria migrate to the final destination, which can be the spleen, heart, bones, brain, or the reproductive system, where they colonize the tissue (6). Colonization of the trophoblastic tissue of the placenta is often, but not always, concomitant with abortion in pregnant animals (6). Material from abortions, namely placenta, fetus, fetal fluids, and vaginal discharges are highly infectious with high loads of bacteria (14). The excretion of *Brucella* in vaginal discharge can continue for up to two months following delivery or abortion and may also be found in milk, urine, semen, and feces (8). Asymptomatic, but infected female animals, can also shed the organism in milk and uterine discharges after delivery (8).

*B. melitensis* can be diagnosed by microscopic examination of stained smears or by culture from fetal membranes, fetal stomach contents, vaginal swabs, semen, or by serology (15). Definitive diagnosis is normally done by isolation and identification of the causative agent, which is time-consuming and hazardous and must be performed by highly skilled personnel. For these reasons, serological tests are normally preferred. In control programs, like the one described below, the surveillance is performed by an annual serological screening of a large number of blood samples from sheep and goats. Genetic and immunological evidence suggests that all members of the genus *Brucella* are closely related. Subsequently, serological tests are not always completely specific and they can also cause cross-reactions to other bacteria, especially *Yersina enterocolitica* serotype O:9 (16). The humoral immune response to *Brucellae* is dominated by antibodies to the smooth lipopolysaccharide (S-LPS) of *Brucella* spp. (17). The antibody response shows a typical IgM/IgG shift with time after infection (18). Cross-reactivity has been observed between *Brucella* S-LPS and S-LPS of other bacteria, for example, *Francisella tularensis*, *Escherichia coli* O:157 and O:116, and *Y. enterocolitica* O:9. Especially *Y. enterocolitica* O:9 possesses an obstacle in the diagnostics, since the immunodominant O-chain of S-LPS of *Y. enterocolitica*, serotype O:9, and *Brucella* spp. are identical (19).

Rose Bengal Test (RBT) and the Complement Fixation Test (CFT) are some of the most commonly used serological tests, which are also recommended by OIE (20) as prescribed tests for international trade. They are both based on the detection of antibodies to a solubilized *B. abortus* antigen. The value of these tests in truly infected sheep has been questioned, since sensitivities vary between approximately 90 and 100%, being more predictable in acute phases of brucellosis than in chronic disease (21). The RBT is a rapid slide-type agglutination assay performed with a stained *B. abortus* suspension. It is simple to perform, but sensitivity, especially in long evolution, is lowered because of blocking IgA antibodies or presence of non-agglutinating antibodies. RBT detects S-LPS-specific IgM, IgG, and IgA (18). The CFT is a more complex test, where antibodies are demonstrated by agglutination when complement is attached to an antigen-antibody complex in the presence of a system with sensibilized sheep erythrocytes.

### Y. enterocolitica

*Y. enterocolitica* serotype O:9 is one of the human pathogenic *Y. enterocolitica* serotypes, which can also infect animals (22), generally without causing any symptoms. Pigs, sheep, and cattle can be carriers of pathogenic serotypes of *Y. enterocolitica* in their intestinal flora, and pigs are well known reservoirs of *Y. enterocolitica* in their tonsils (23–25). Prevalence of *Y. enterocolitica* seem to be on the rise, at least in cattle, as it was rarely seen before the 1990s and since then has been regularly isolated within the European Union (EU) and also other parts of the world such as New Zealand (11). *Y. enterocolitica* O:9 can give rise to false**-**positive results in serological tests for brucellosis due to their identical O-antigen structure with *Brucella* spp. (13). *Y. enterocolitica* O:9 can be detected in healthy pigs, and according to OIE, none of the serological tests for brucellosis can be reliably used to diagnose individual pigs due to the positive reactions caused by *Y. enterocolitica* O:9 (14). Identification of *Y. enterocolitica* serotype O:9 can be carried out using culture followed by slide agglutination and a panel of biochemical tests. However, there exists non-pathogenic *Y. enterocolitica* O:9 strains. If pathogenicity is to be determined, the biotype of a strain (and not only the serotype) can be demonstrated. Alternatively, the virulence potential of an *Y. enterocolitica* O:9 isolate can be shown. This can be carried out, for example, by examination for presence/absence of the chromosomally located gene *ail* by PCR (26) and by stating presence/absence of the *Y. enterocolitica* virulence plasmid (27).

## The Swedish surveillance program for *B. melitensis*

Brucellosis, an OIE-listed disease with severe zoonotic implications, has never been diagnosed in Swedish sheep or goats. Brucellosis in cattle was eradicated from the Swedish cattle population during the first half of the last century, and the last Swedish bovine case was recorded in 1957. Sweden's bovine brucellosis-free status is officially stated in EU legislation since 1994 (Decision 2003/467/EC), and Sweden was declared officially free of brucellosis in sheep and goats in 1995 (Decision 94/972/EC). Brucellosis in food-producing animals is included in the Swedish Act of Epizootics (SFS 1999:657 with amendments). Vaccination according to this law is prohibited and notification of suspected cases is mandatory. Current surveillance standards for brucellosis in sheep and goats are given in the EU legislation, Directive 91/68/EEC. Screening for brucellosis in sheep and goats have been regularly conducted in Sweden since 1995 with approximately 10,000 samples tested each year, representing approximately 5% of the sheep population. The purpose of the surveillance is to document freedom from brucellosis in sheep and goats in Sweden, in accordance to Directive 91/68/EEC. The Swedish Board of Agriculture finances this surveillance program, which is planned and performed by the National Veterinary Institute, SVA. All diagnostic testing is performed at SVA. The diagnostic test in the program is the RBT. In case of positive reaction with the RBT, the result is confirmed with CFT. If these tests are both positive, the herd is considered a suspected case of *B. melitensis* and further testing and epidemiological investigations are performed. During 2011, 7,141 serum samples from 764 sheep flocks and 301 serum samples from 41 goat flocks were analysed for *B. melitensis* in the surveillance program (28).

## Case description

### Sampling and results

In November 2011, three samples out of ten, originating from a herd with 20 sheep, tested positive in the RBT within the surveillance program for *B. melitensis*. The results were confirmed with the CFT. Since the CFT results were also positive, the herd was considered a suspected case of *B. melitensis* and put under restrictions, including movement of animals for sale and slaughter. Epidemiological investigation revealed that the herd had had neither clinical symptoms coherent with brucellosis nor any known contacts that presumably could have introduced the infection. The last animal introduced was a ram in 2010. The herd from where the ram originated had not experienced any symptoms of brucellosis. All 20 sheep were resampled three weeks later. In this sampling, two out of the first three positive sheep plus three other sheep tested positive in CFT (see Table 1). In parallel with the second serological sampling, individual fecal samples were taken from all sheep in the herd and the samples were cultured for *Y. enterocolitica*. Five out of 20 fecal samples were found positive for *Yersinia* spp. Four of these were confirmed as *Y. enterocolitica* serotype O:9 (see Table 2). The entire herd was again serologically tested in January 2012, five weeks after the second sampling. All sera were tested both with the RBT and the CFT. This time, all results were negative for both tests. On receiving the negative results from the third serological testing, all restrictions for the herd were terminated. As for the previous lambing season, the lambing-results of 2012 were normal and no symptoms related to possible infection with *B. melitensis* were observed.

**Table 1:** Serological results from a herd of sheep within the Swedish surveillance program for *Brucella melitensis* and follow-up samples from the same herd

	22.11.2011		16.12.2011		25.01.2012	
			
Animal/date of sampling	RBT	CFT IU/ml	RBT	CFT IU/ml	RBT	CFT IU/ml
2	neg	–	pos	80	neg	neg
3 (ram)	–	–	neg	neg	neg	neg
4	pos	160^1^	neg	40	neg	neg
6	pos	80^1^	neg	neg	neg	neg
8	neg	–	neg	neg	neg	neg
10	neg	–	neg	neg	neg	neg
12	neg	–	neg	neg	neg	neg
16	neg	–	neg	neg	neg	neg
22	neg	–	neg	neg	neg	neg
38	neg	–	neg	neg	neg	neg
40	pos	80^1^	neg	80	neg	neg
96	–	–	neg	neg	neg	neg
98	–	–	neg	neg	neg	neg
100	–	–	neg	neg	neg	neg
102	–	–	neg	neg	neg	neg
104	–	–	pos	80	neg	neg
106	–	–	neg	neg	neg	neg
108	–	–	neg	neg	neg	neg
110	–	–	neg	neg	neg	neg
112	–	–	pos	80	neg	neg

Pos, positive; neg, negative.

On the first occasion, Rose Bengal Test (RBT) was performed on all tested sera and Complement Fixation Test (CFT) was performed only on sera positive in RBT.

1 Indicated samples were confirmed positive in CFT by the EU Reference laboratory (EU-RL) for brucellosis.

**Table 2:** Results of culturing of fecal samples for *Yersinia* spp. and typing of isolates in 20 sheep

Animal/date of results	Direct culture 26.12.2011	Enrichments in PSB and MRB 4.1.2012	Cold enrichment in PSB for 3 weeks 14.1.2012	Cold enrichment for 3 weeks manure 14.1.2012	Serotype/biotype
2^1^	neg	neg	neg	neg	
3 (ram)	neg	neg	neg	neg	
4^1^	neg	neg	neg	neg	
6^1^	neg	neg	neg	neg	
8	neg	pos	pos	neg	*Y. rohdei* biotype O:13,7
10	pos	pos	pos	pos	*Y. enterocolitica* O:9/biotype 2
12	neg	neg	neg	neg	
16	neg	neg	neg	neg	
22	neg	neg	neg	neg	
38	neg	neg	neg	neg	
40^1^	neg	neg	neg	neg	
96	neg	neg	neg	neg	
98	neg	neg	neg	neg	
100	neg	neg	neg	neg	
102	neg	neg	neg	neg	
104^1^	neg	neg	neg	neg	
106	neg	neg	neg	neg	
108	pos	pos	pos	neg	*Y. enterocolitica* O:9/biotype 2
110	pos	pos	pos	neg	*Y. enterocolitica* O:9/biotype 2
112^1^	Neg	pos	pos	neg	*Y. enterocolitica* O:9/biotype 2

Pos, positive; neg, negative.

1 Six sheep were shown to be serologically positive for *Brucella melitensis* in a sampling adjacent or parallel in time with sampling for *Yersinia* spp.

### Results from further characterization of Y. enterocoloitica serotype O:9

Professor Wauters at the Université Catholique de Louvain in Brussels confirmed the strain characterization by identifying the same four strains to *Y. enterocolitica* serotype O:9. In addition, Wauters performed biotyping of the strains and found them to belong to biotype 2. Wauters also examined the pathogenicity of the strains, that is, performed phenotypic tests using absorbed specific antisera against the YadA protein (protein P1) linked to the virulence plasmid, and the myf protein (mucoid *Yersinia* factor) present in potential pathogenic strains but not depending on the virulence plasmid. The four biotype 2/serotype 9 strains were positive in both tests. This correlated to the virulence test results obtained at the National Food Agency in Sweden. The non-serotype O:9 isolate was identified to *Y. rohdei* biotype O:13,7.

### B. melitensis serology

Brucella RBT (Bio-Rad, Marnes-la-Coquette, France) was performed according to the producer's instruction. Samples showing any degree of agglutination in the RBT within four minutes were considered as positive and subsequent analysis by CFT was performed. The CFT was performed by the cold procedure as described in the manual (Standard Operating Procedure, revision 3, 2010) from the EU Reference Laboratory (EU-RL) for brucellosis at Anses in Maisons-Alfort Cedex, France. The system was standardized to the *B. abortus* positive International Standard Antiserum, OIEISS (Brucella Reference Centre, AHVLA, Weybridge, United Kingdom), allowing the direct transformation of a given endpoint, that is, the highest dilution showing 25% inhibition of haemolysis, to the titer in International units. Sera giving a titer equivalent to 20 IU/ml or more were considered to be positive. Samples positive in the CFT from the first sampling were confirmed by the EU-RL for brucellosis.

### Culturing of Y. enterocolitica

The method for detecting *Y. enterocolitica* was determined qualitatively by a combination of cold enrichment, selective enrichment, and subculture on selective agar media. The fecal samples were mixed with phosphate-buffered saline containing 2% sorbitol and 0.15% bile salts (PSB) in a dilution of 1:10. The remaining sample materials were stored at 4°C for three weeks. The samples in PBS were incubated at 20–25°C (room temperature) for three hours, after which an aliquot was inoculated onto a selective agar medium; Cefsulodin-Irgasan-Novobiocin agar (CIN agar). The CIN-agar was incubated at 30°C for 24 hours. The rest of the inoculated PSB broth was enriched at 4°C. When the cold enrichment cultures were 8 days old, 0.1 ml was taken for selective enrichment in a modified Rappaport broth (MRB) at 20–25°C for four days. The remaining PSB broth was incubated again at 4°C for further two weeks. After four days, an aliquot from MRB was streaked onto CIN-agar and incubated at 30°C for 24 hours. MRB is a selective broth, favoring the growth of serogroups O:3 and O:9 and inhibiting most other bacteria or other *Yersinia* variants. After three weeks, the PSB were thoroughly mixed and then an aliquot was streaked onto CIN-agar and incubated at 30°C for 24 hours. A sample from the remaining manure, which had been stored at 4°C, was also streaked onto CIN-agar and incubated at 30°C for 24 hours. Suspected colonies from the CIN agar were sub-cultured onto horse blood agar and incubated at 37°C for 24 hours. All substrates were produced in-house according to the Nordic Committee on Food Analysis (29).

### Characterization of Y. enterocolitica

Gram staining and biochemical test (API 20E, Biomérieux, Frankrike) were performed on suspected strains. Strains identified as *Y. enterocolitica* were tested using antisera against *Y. enterocolitica* O:3 and O:9 (Reagensia AB, Sweden). Four out of the five examined *Y. enterocolitica* isolates agglutinated with the O:9-serum, that is, corresponded to *Y. enterocolitica* serotype O:9. The fifth isolate did not agglutinate by serum O:9. The four serotype O:9 isolates also gave positive PCR results when analyzed using a TaqMan probe-based real-time polymerase chain reaction (PCR) method targeting the *ail* gene (20). The non-serotype O:9 isolate gave a negative PCR result. In addition, the same four isolates produced pinpoint colonies while growing on CR-BHO medium indicating the presence of the *Y. enterocolitica* virulence plasmid (27). Further characterization was carried out as described above.

## Discussion

Ever since the start of using serology for diagnosing brucellosis more than 100 years ago, it has been realized that this type of test is prone to false**-**positive reactions, resulting from, for example, exposure to other micro-organisms (30). Many gram-negative bacteria, such as *Vibrio cholerae* O1, *E. coli* O:157, *Escherichia hermani*, *Stenotrophomonas maltophilia*, *Salmonella group* N, and *Y. entercolitica* O:9, have been incriminated for causing false**-**positive reactions, but only *Y. entercolitica* O:9 seems to cause a reaction, which is strong and persistant and thus interferes with the diagnosis of brucellosis (31, 32). In an experimental oral infection of heifers with *Y. enterocolitica* O:9, Garin-Bastuji et al. (16) demonstrated a positive serological result for *Brucella* spp. in two out of eight animals in RBT and CFT during the 78-day experimental period. In one of the animals, a positive reaction was demonstrated in 16 out of 21 samplings and in the other in 1 and 2 samplings (RBT and CFT, respectively), showing large individual differences. All animals excreted *Y. enterocolitica* on one or more occasions during the experimental period. It has also been shown by Godfroid et al. (13) that none of the serological tests commonly used manage to differentiate infections with brucellosis from those with *Y. entercolitica* O:9. According to Godfroid et al. (13), only the brucellosis skin test, not described here and not commonly used, is specific. Reports of infections with *Y. entercolitica* O:9 interfering with control programs for brucellosis in bovines are numerous (13, 31), the phenomena especially causing problems for countries at the end of the eradication program or for countries officially free from brucellosis, such as Sweden (13). This is due to the fact that the positive predictive value (the probability of a test positive animal to be truly infected) decreases more and more with decreasing assumed prevalence, causing higher levels of test positives to be expected to be false positives as the prevalence reaches zero. Godfroid and Käsbohrer (11) highlight the increasing prevalence of infection with *Y. entercolitica* O:9 since the 1990s as one out of three factors seriously hampering eradication of brucellosis in Europe. Yet, despite the imperfect serological tests and the presence of *Y. entercolitica* O:9 interfering with the results, the absence of brucellosis from a herd or a country can be proven – by in-depth epidemiological investigations and the absence of isolation of *Brucellae* from positive animals (13).

Despite Sweden's relatively long history of testing sheep and goats serologically for presence of antibodies against *B. melitensis*, false**-**positive reactions have not previously been a problem (28). Solitary positive reactions have occasionally occured, but until the case described here neither several in the same herd, nor with antibody titers to a level that would suggest true infection with *B. melitensis*. The prevalence of *Y. entercolitica* O:9 in sheep in Sweden is unknown. However, recent studies suggest that it is low as no bacteria could be cultured from 99 sheep investigated from one of the areas with the highest density of sheep in Sweden (33). Hilbink et al. (31) observed the same phenomena in New Zealand in 1992 when 35 out of 1,071 deer in an export consignment reacted positively with the RBT for *B. abortus*. *B. abortus* was at the time just eradicated from the country, and such false**-**positive reactions had not previously been seen. *Y. entercolitica* O:9 was cultured from one of the deers. Shortly after, false**-**positive reaction toward *B. abortus* started to be common also in cattle herds, with *Y. entercolitica* O:9 being identified as the cause. Whether the results presented here, with the first identification of *Y. entercolitica* O:9 as the cause of false**-**positive reactions for *B. melitensis* in Swedish sheep, is an exception or the beginning of a rising prevalence of infections with *Y. entercolitica* O:9 in sheep in Sweden, as previously seen for cattle and pigs in other parts of the world, remains to be seen. As the Swedish surveillance program for *B. melitensis* in sheep and goats continues, any changes in the prevalence of agents causing false**-**positive reactions will be well registered and reported. An increase in the prevalence of *Y. entercolitica* O:9 might cause problems as previously described in other countries. Epidemiological investigation and culture of bacteriae will be used also in the future to rule out any suspicious cases.
